# Polyscore of autonomic parameters for risk stratification of the elderly general population: the Polyscore study

**DOI:** 10.1093/europace/euaa359

**Published:** 2020-12-04

**Authors:** Alexander Steger, Michael Dommasch, Alexander Müller, Daniel Sinnecker, Katharina M Huster, Teresa Gotzler, Othmar Gotzler, Alexander Hapfelmeier, Kurt Ulm, Petra Barthel, Katerina Hnatkova, Karl-Ludwig Laugwitz, Marek Malik, Georg Schmidt

**Affiliations:** 1 Klinik für Innere Medizin I, Technische Universität München, Ismaninger Str. 22, 81675 Munich, Germany; 2 INVADE Study Group, Karl-Böhm-Str. 32, 85598 Baldham, Germany; 3 Institute of Medical Informatics, Statistics and Epidemiology, Technische Universität München, Grillparzerstr. 18, 81675 Munich, Germany; 4 National Heart and Lung Institute, Imperial College, ICTEM, Hammersmith Campus, 72 Du Cane Road, Shepherd's Bush, London W120NN, UK; 5 Department of Internal Cardiology Medicine, Faculty of Medicine, Masaryk University, Jihlavská 20, 625 00 Brno, Czech Republic

**Keywords:** Elderly general population, Prospective validation, Autonomic markers, Risk assessment, Polyscore

## Abstract

**Aims:**

Present society is constantly ageing and elderly frequently suffer from conditions that are difficult and/or costly to treat if detected late. Effective screening of the elderly is therefore needed so that those requiring detailed clinical work-up are identified early. We present a prospective validation of a screening strategy based on a Polyscore of seven predominantly autonomic, non-invasive risk markers.

**Methods and results:**

Within a population-based survey in Germany (INVADE study), participants aged ≥60 years were enrolled between August 2013 and February 2015. Seven prospectively defined Polyscore components were obtained during 30-min continuous recordings of electrocardiogram, blood pressure, and respiration. Out of 1956 subjects, 168 were excluded due to atrial fibrillation, implanted pacemaker, or unsuitable recordings. All-cause mortality over a median 4-year follow-up was prospectively defined as the primary endpoint. The Polyscore divided the investigated population (*n* = 1788, median age: 72 years, females: 58%) into three predefined groups with low (*n* = 1405, 78.6%), intermediate (*n* = 326, 18.2%), and high risk (*n* = 57, 3.2%). During the follow-up, 82 (4.6%) participants died. Mortality in the Polyscore-defined risk groups was 3.4%, 7.4%, and 17.5%, respectively (*P* < 0.0001). The Polyscore-based mortality prediction was independent of Framingham score, diabetes, chronic kidney disease, and major stroke and/or myocardial infarction history. It was particularly effective in those aged <75 years (*n* = 1145).

**Conclusion:**

The Polyscore-based mortality risk assessment from short-term non-invasive recordings is effective in the elderly general population, especially those aged 60–74 years. Implementation of a comprehensive Polyscore screening of this age group is proposed to advance preventive medical care.

**What’s new?**

- The Polyscore is a combination of seven predominantly autonomic risk markers assessed in simultaneous non-invasive short-term recordings of electrocardiogram, blood pressure, and respiration.
- Previously, the Polyscore has been shown to be a strong mortality risk predictor in survivors of acute myocardial infarction.
- Presently, the Polyscore was prospectively validated as a strong mortality risk predictor in the elderly general population aged ≥60 years dividing the population into low, intermediate, and high-risk groups.
- The Polyscore is independent of other conventional risk factors and scores. It is not independent of age and particularly effective among individuals aged 60–74 years.
- Implementation of a comprehensive Polyscore screening in this age group is proposed to advance preventive medical care.

## Introduction

The constantly ageing society of the western world^1^ raises the importance of general risk prediction for the elderly.^2^ While many subjects reach old age in relatively good health conditions, others suffer from a variety of undiagnosed ailments. If these conditions are detected late only after the clinical condition worsens, they are frequently difficult and/or costly to treat. At the same time, however, subjects of moderately advanced age are presently too numerous to be regularly invited for a thorough and comprehensive medical examination.

It is therefore important to identify those elderly subjects who need to be examined more closely. This is well known, and various assessment systems have already been developed and integrated into health practices.^3^ While such assessment systems contribute to the risk stratification of the general population, they are less effective in the elderly.^4^

Since the autonomic nervous control and reflexes maintain the homeostasis of the organism, it is reasonable to expect that their characterization would provide strong prognostic information, thus allowing individual risk assessment. Here, we present a prospective risk prediction study in a non-selected general population of retirement age or on the transition to the retirement age. The aim of this validation study was to prospectively test a novel risk predictor consisting of seven predominantly autonomic tests, the Polyscore.^5^ The Polyscore has originally been developed in a population of patients who survived the acute phase of myocardial infarction. Consistent with the original design, the Polyscore was assessed non-invasively in a 30-min session during a regular visit to a general practitioner.

## Methods

### Population

The study was a prospectively set-up sub-study of the INVADE (Intervention Project on Cerebrovascular Disease and Dementia in the District of Ebersberg) population-based prevention investigative study. As previously published,^6^ the INVADE study was based on primary care in the catchment area of Ebersberg in Upper Bavaria, Germany, and aimed at systematic identification and evidence-based treatment of cardiovascular risk factors in an elderly population. The study was supported by Allgemeine Ortskrankenkasse (AOK) Bayern, the largest Bavarian health insurance. All insured subjects aged ≥60 years within the INVADE study catchment area were invited to participate in the investigation.

The Polyscore study was designed to validate the Polyscore^5^ prospectively in an elderly general population. Between August 2013 and February 2015, altogether 1956 participants accepted the invitation to participate and were included in the Polyscore study. The study complied with the Declaration of Helsinki was approved by the local ethics committee and all participants gave written informed consent.

### Investigations

All participants attended a baseline visit during which simultaneous non-invasive 30-min recordings were made. These included an electrocardiogram (ECG) (5-electrode-ECG with electrode positions of right atrium, left atrium, LF, V1, and neutral, from which orthogonal leads XYZ are obtained, sampled at 300 Hz), continuous arterial blood pressure (finger photoplethysmography, sampled at 200 Hz), and chest impedance (sampled at 300 Hz). The signal acquisitions (NOVA system, Finapres Medical Systems B.V., Enschede, the Netherlands) were conducted in supine resting position in a noiseless undisturbed environment. All collected signals were pseudonymized. Artefact elimination, QRS complex morphology classifications (normally conducted beats, conduction defects or ventricular ectopic beats), and the Polyscore calculation were performed automatically. The results were reviewed and if necessary corrected by an experienced technician blinded to the clinical outcome data, which in general did not take more than 2 min per recording. Only participants who presented in sinus rhythm (i.e. without atrial fibrillation or implanted pacemaker) were included in further analyses.

### Polyscore definition

As previously described in an independent study (ART study) including patients who survived a myocardial infarction,^5^ the collected signals were used to determine seven primarily autonomic risk factors. Each of these seven risk factors, derived from the same 30-min recordings, was evaluated in each study participant and classified as normal or abnormal. Dichotomy limits of the individual risk parameters were predefined based on the results of the ART study and prospectively applied in this validation study. No retrospective adjustments or manipulations of the dichotomy limits were allowed:

1. Heart rate turbulence slope that is the gradual development of RR intervals following a ventricular premature contraction, dichotomized at 2.5 ms per RR interval.^7^ For the calculation of heart rate turbulence slope, the presence of one single premature ventricular ectopic beat is sufficient. The absence of ectopic beats is generally considered as prognostically favourable. Therefore, consistent with the established heart rate turbulence standards, these cases were handled as having the parameters normal (0 points for HRT);
2. Deceleration capacity quantifying vagal effects by assessing deceleration-related heart rate modulations, dichotomized at 2.5 ms average beat-to-beat RR-interval prolongation^8^;
3. Baroreflex sensitivity, assessed by bivariate phase-rectified signal analysis of blood pressure rise and simultaneous RR interval changes, dichotomized at 1.58 ms per mmHg^9^;
4. Average respiration rate derived from the chest impedance signals, dichotomized at 18.6 breaths per minute^10^;
5. Expiration-triggered sinus arrhythmia, assessed by bivariate phase-rectified signal analysis of RR interval changes during the early expiration phase, dichotomized at 0.19 ms of RR interval change^11^;
6. Post-ectopic systolic blood pressure potentiation, expressed as the ratio of the systolic blood pressure of the first post-ectopic beat relative to the systolic pressure values of the following sinus rhythm cycles, dichotomized at 1.03.^12^ Similar to HRT, recordings without any ventricular ectopic beats were considered to have the parameters normal; and
7. Increased ectopic frequency, defined as the presence of ≥7 supraventricular or ≥29 ventricular ectopic complexes during the 30-min recordings.^5^

After evaluation, these seven risk factors were combined into a Polyscore of eight possible values ranging between zero (all risk factors normal) and seven (all risk factors abnormal). In the ART study, three risk strata defined as Polyscore 0–2 (low risk), 3–4 (intermediate risk), and 5–7 (high risk) were established.^5^

In the present study, the risk factors defining the Polyscore, their dichotomy limits, and three predefined risk strata were applied prospectively without any modifications.

### Modified Framingham score

Conventional cardiovascular risk assessment was performed according to a modification of the Framingham score^13^ calibrated to the German setting as broadly applied by German general practitioners (http://www.arriba-hausarzt.de).^14^ Briefly, the algorithm includes sex, age, current smoking status, total cholesterol, HDL cholesterol, systolic blood pressure, antihypertensive medication use, family history of cardiovascular disease, clinical evidence of diabetes mellitus with HbA1c in diabetic patients, and clinical evidence of arteriosclerosis. The individual risk profile is quantified by a numerical score that can be converted into a 10-year risk for cardiovascular events in percent. Within the present Polyscore study, the modified Framingham score was dichotomized at ≥20% to identify high-risk individuals. This cut-off was predefined and corresponds to the present clinical practice.

### Follow-up

All-cause mortality was the primary, prospectively defined clinical endpoint of the study. Participants were followed-up over a median period of 4.0 years [interquartile range (IQR) 3.6–4.3 years]. There were no patients lost during the follow-up. Participants who did not adhere to a 3-month follow-up schedule were contacted by letter or by telephone. If this was not successful, the local population register was used to ascertain the survival status of the patient.

On study termination in February 2018, all patients reached either the primary endpoint or the prospectively established minimum follow-up period of 30 months. Hence on study termination, all surviving patients were censored. The follow-up duration of the first and the last enrolled patient was 4.48 and 3.01 years, respectively.

### Mortality prediction

As stated, the study was prospectively set-up to investigate the power of the Polyscore to predict mortality in the elderly general population. Consequently, the study assessed the Polyscore independence of the conventional cardiovascular risk factors and of the modified Framingham score.

Since the Polyscore is primarily based on a battery of autonomic tests and since autonomic reactions are severely affected by diabetic neuropathy,^15^ the investigation of the predictive power of the Polyscore was repeated separately for participants suffering and not suffering from diabetes mellitus. Autonomic responsiveness is also reduced by advanced age.^16^ Consequently, the Polyscore was investigated separately in participants below and above the age of 75 years.

### Statistics

The distribution of quantitative data is presented by median and IQR. Qualitative data is described by absolute and relative frequencies. Log-rank tests were used for hypothesis testing of differences between group-specific Kaplan–Meier estimates of survival. Marginal and conditional effect estimates of variables are given by the hazard ratio (HR) and were assessed by univariable and multivariable Cox proportional hazards regression models, respectively. The discriminatory power of the regression models was quantified by the concordance index (c-index). The likelihood-ratio test was used to compare nested models. Additional multivariable models, each with a main effect of a binary variable, which was used to define two subgroups, a main effect of the Polyscore classes and a corresponding interaction effect were computed for the purpose of subgroup analyses. Hypothesis testing of the interaction effect was used to infer on significantly different effects of the Polyscore in the investigated subgroups. Hypothesis testing was generally performed on exploratory two-sided 5% significance levels. Analyses were conducted using IBM SPSS Statistics version 25 (IBM Corp., Armonk, NY, USA).

Similar to the Plyscore definition and risk grouping, the described statistical analyses were defined prospectively.

## Results

**Figure 1:**
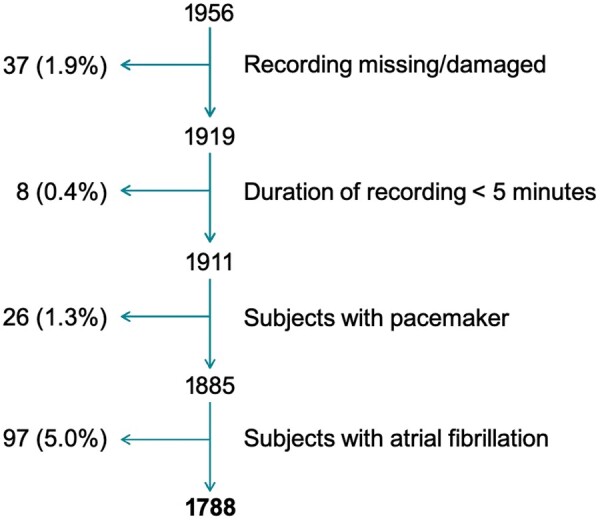
Study flow chart.

Non-invasive 30-min recordings of an ECG, arterial blood pressure, and chest impedance were obtained in all 1956 study participants. Of these, 168 (8.6%) were excluded because of atrial fibrillation (*n* = 97, 5.0%), implanted pacemaker (*n* = 26, 1.3%) and technically unsuitable recordings (*n* = 45, 2.3%). The remaining 1788 subjects constituted the investigated study population (*Figure 1*).

**Figure 2:**
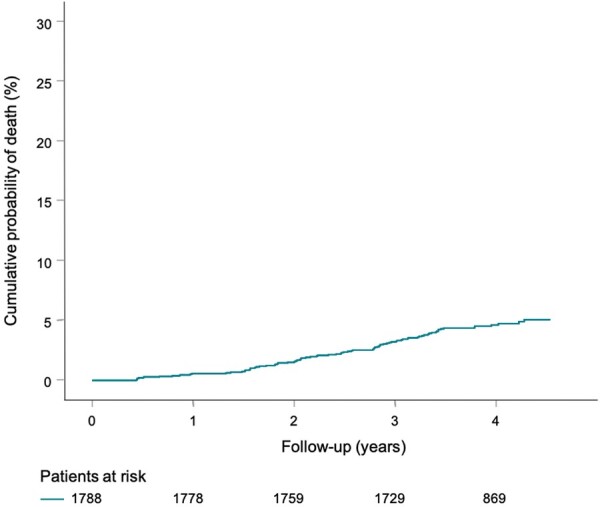
All-cause mortality in the study population. The Kaplan–Meier curve of probability of death is shown. Numbers of participants at risk are shown below the time axis.

Clinical characteristics of the study population are shown in *Table 1*. Mean age at inclusion was 72 years and the proportion of women was 58%. Of the 1788 study participants, 82 (4.6%) died during a median follow-up of 4.0 years (IQR 3.6–4.3 years). As expected in a general population, the death incidence was almost uniformly distributed, and the slope of the corresponding Kaplan–Meier curve was essentially linear (*Figure 2*, *Table 1*).

**Table 1:** Clinical characteristics of the study population

Variable	*n* = 1788
Age, years	72 (67–76)
Age ≥ 75 years, *n*	643 (36%)
Females, *n*	1032 (58%)
BMI, kg/m^2^	28 (25–31)
History of CAD, *n*	257 (14%)
History of PAD, *n*	153 (9%)
History of stroke, *n*	118 (7%)
Diabetes mellitus, *n*	639 (36%)
HbA1c, %	5.7 (5.4–6.1)
Renal insufficiency, *n*	174 (10%)
eGFR, mL/min	72 (59–85)
Hypertension, *n*	1376 (77%)
Non-smoking, *n*	1274 (71%)
Hyperlipidaemia, *n*	1045 (58%)
Total cholesterol, mg/dL	207 (182–239)
Depression, *n*	293 (16%)
Mod. Framingham score, %	14.5 (8.1–24.1)
ACE-inhibitors, *n*	787 (44%)
AT1-blockers, *n*	349 (20%)
Ca-antagonists, *n*	366 (21%)
β-blockers, *n*	684 (38%)
Diuretics, *n*	739 (41%)
Statins, *n*	696 (39%)
Antidepressants, *n*	196 (11%)
All-cause mortality, *n*	82 (4.6%)
Follow-up time, years	4.0 (3.6–4.3)

Incidence variables are shown as absolute number (%), numerical variables as median (interquartile range).

ACE, angiotensin converting enzyme; AT1, angiotensin 1; BMI, body mass index; CAD, coronary artery disease; eGFR, estimated glomerular filtration rate; HbA1c, haemoglobin A1c; PAD, peripheral artery disease.

**Figure 3:**
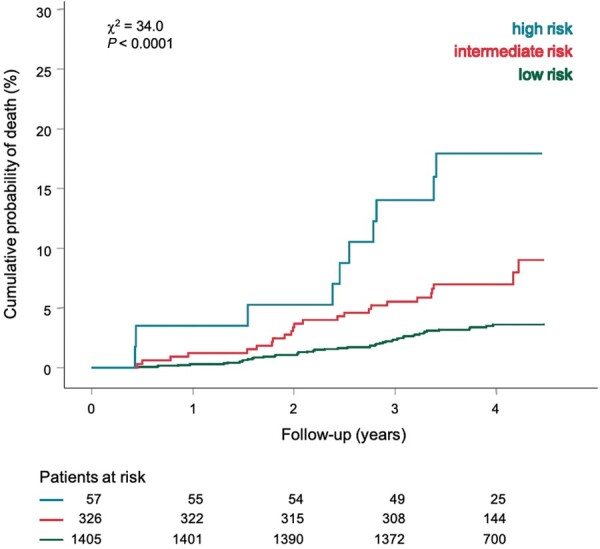
Kaplan–Meier probabilities of death according to three Polyscore risk strata: low (green), intermediate (red), and high risk (turquoise). Numbers of participants at risk in the individual subgroups are shown below the time axis. *χ*^2^, chi-square.

The Polyscore was classified as low risk in 1405 (78.6%), intermediate risk in 326 (18.2%), and high risk in 57 (3.2%) participants. The observed mortality rates within these groups were 3.4%, 7.4%, and 17.5%, respectively. The difference between the cumulative probabilities of death in these groups was statistically significant (*χ*^2^ 34.0, *P* < 0.0001). Thus, a large group at very low mortality risk and a small group at particularly high mortality risk were identified. Kaplan–Meier probability of death curves for the three Polyscore risk strata are shown in *Figure 3*. Interestingly but not perhaps surprisingly, the Polyscore predicted all-cause mortality but did not predict malignancy-related mortality as shown in Supplementary material online, *Figure S1*.

In a univariable Cox regression analysis, the Polyscore was a strong predictor of outcome. Compared to the low-risk stratum, intermediate-risk Polyscore was associated with an HR of 2.24 [95% confidence interval (CI) 1.37–3.65] and high-risk Polyscore with an HR of 5.55 (95% CI 2.81–10.97). Age, diabetes, chronic kidney disease, and history of major stroke also predicted all-cause mortality, whereas prediction by the history of myocardial infarction was not statistically significant (*Table 2*). A multivariable analysis comprising all these confounders confirmed the highly significant association of the Polyscore with all-cause mortality (intermediate risk: HR 1.80, 95% CI 1.09–2.96; high risk: HR 4.01, 95% CI 1.99–8.12) (*Table 2*). Another multivariable model including the modified Framingham risk score, as already described (HR 2.19, 95% CI 1.41–3.40) and the Polyscore (intermediate risk: HR 2.01, 95% CI 1.22–3.29; high risk: HR 4.53, 95% CI 2.27–9.04) clearly demonstrated additive value of both predictors (*Table 3*). Upon addition of the Polyscore to the modified Framingham score in a multivariable approach, the concordance index improved significantly from 0.616 to 0.669 (*χ*^2^ 18.4, *P* < 0.0001).

**Table 2:** Univariable and multivariable Cox regression models using dichotomized variables in all patients

Variable	Univariable model			Multivariable model		
	HR	*χ* ^2^	*P*-value	HR	*χ* ^2^	*P*-value
Age ≥ 75 years	2.47 (1.59–3.82)	16.3	<0.0001	2.07 (1.33–3.23)	10.4	0.001
Diabetes (y/n)	1.58 (1.02–2.44)	4.3	0.039	1.13 (0.71–1.78)	0.3	0.610
Chronic kidney disease (y/n)	2.93 (1.76–4.90)	16.9	<0.0001	2.14 (1.24–3.69)	7.5	0.006
History of AMI (y/n)	1.69 (0.82–3.51)	2.0	0.157	1.12 (0.53–2.37)	0.1	0.758
History of major stroke (yes/no)	2.49 (1.35–4.59)	8.5	0.004	1.69 (0.90–3.17)	2.7	0.103
Polyscore intermediate risk	2.24 (1.37–3.65)	10.4	0.001	1.80 (1.09–2.96)	5.3	0.022
Polyscore high risk	5.55 (2.81–10.97)	24.3	<0.0001	4.01 (1.99–8.12)	15.0	<0.0001

AMI, acute myocardial infarction; HR, hazard ratio; n, no; y, yes; *χ*^2^, chi-square.

**Table 3:** Univariable and multivariable Cox regression models including the dichotomized modified Framingham score in all patients

Variable	Univariable model			Multivariable model		
	HR	*χ^2^*	*P*-value	HR	*χ^2^*	*P*-value
Mod. Framingham Score ≥ 20 %	2.53 (1.64–3.90)	17.5	<0.0001	2.19 (1.41–3.40)	12.1	0.001
Polyscore intermediate risk	2.24 (1.37–3.65)	10.4	0.001	2.01 (1.22–3.29)	7.6	0.006
Polyscore high risk	5.55 (2.81–10.97)	24.3	<0.0001	4.53 (2.27–9.04)	18.4	<0.0001

HR, hazard ratio; *χ*^2^, chi-square.

**Figure 4:**
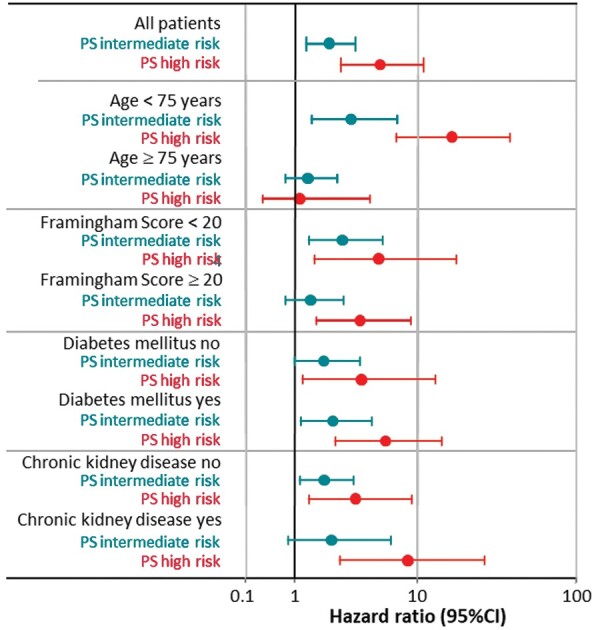
Subgroup analysis. Relative mortality risks (hazard ratios with 95% confidence intervals) in participants with intermediate risk (turquoise) and with high-risk (red) Polyscore values in comparison to those with low-risk Polyscore values in the total population and in different subgroups. CI, confidence interval; PS, Polyscore.

**Figure 5:**
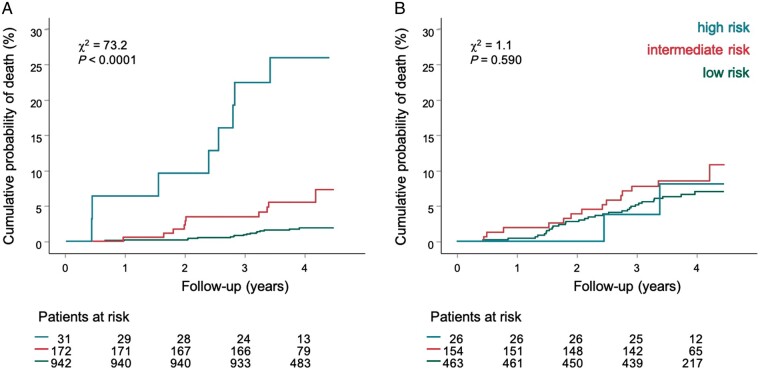
Age. Comparison of Kaplan–Meier probabilities of death in participants aged 60–74 years (*A*) and in participants aged ≥75 years (*B*) at study inclusion according to three Polyscore risk strata: low (green), intermediate (red), and high risk (turquoise). Numbers of participants at risk in the individual subgroups are shown below the time axis. *χ*^2^, chi-square.

Subgroup analyses were performed comparing the HRs of the Polyscore risk strata between patients below and above 75 years of age (*P* < 0.001), with the modified Framingham score below and above 20% (*P* = 0.678), with and without diabetes mellitus (*P* = 0.589), and with and without chronic kidney disease (*P* = 0.271) (*Figure 4*). Of all variables shown in *Figure 4*, only interaction of the Polyscore with age was significant (*χ*^2^ = 18.4, *P* < 0.0001). The statistically significant difference between the participants aged 60–74 years and ≥75 years at inclusion is also documented by Kaplan–Meier probability of death estimates (*Figure 5*). The Polyscore-based mortality prediction was particularly effective in participants aged 60–74 years (*n* = 1145, *χ*^2^ = 73.2, *P* < 0.0001, *Figure 5A*) but not in participants aged ≥75 years (*n* = 643, *χ*^2^ = 1.1, *P* = 0.590, *Figure 5B*). Additionally, both in patients with a Framingham score <20% and in patients with a Framingham score ≥20%, the Polyscore allowed a further separation into three risk strata (Supplementary material online, *Figure S2*).

## Discussion

The study confirms that the Polyscore, a combination of seven autonomic risk factors, is a highly effective predictor of mortality in an unselected population of advanced age. The Polyscore not only characterizes a relatively small group of individuals at a particularly high mortality risk, but it also identifies a large population (about 80% in this cohort) at a noticeably low risk. The Polyscore was found to be particularly strong in individuals of moderately advanced age, i.e. 60–74 years.

The life span increase in the developed world has been substantial over the past decades and this trend is likely to continue. It is naturally welcome that many individuals may now expect not only long life but that they also enjoy good health for years after retirement. Nevertheless, morbidity remains related to age and the overall increased longevity must be considered together with the constantly increasing costs of advanced therapeutic options. Also, the efficacy of many advanced therapeutic possibilities is substantially reduced if they are initiated too late. Therefore, preventive medicinal and healthcare options in the elderly are presently more important than they have ever been before. Targeting preventive medicine successfully requires not only early interventions but also, and equally importantly, identification of those elderly patients in whom preventive and therapeutic interventions are needed.

Every physical examination includes a thorough observation of the patient’s appearance and vital signs, such as pulse rate, respiratory rate, and skin properties (colouring, sweat production, temperature, etc.). The examining physician, thus, recognizes obvious abnormalities of autonomic control that characterize a patient with immediate need of medical support. Modern biosignal analysis also assesses and quantifies autonomic organism control, but it is substantially more sensitive. It can facilitate the identification of individuals at risk suffering from subclinical abnormalities that could lead to the clinical manifestation of disease in future.

We developed a scheme of seven primarily autonomic risk predictors in post-myocardial infarction patients.^5^ We termed their combination ‘the Polyscore’. The tests combined in the Polyscore quantify different autonomic reflexes and organism control mechanisms. Abnormal values signify deviations of the autonomic homeostasis. Coincidence of several abnormalities indicates substantial disorder of the organism, and the risk of fatal complications increases.

In post-myocardial infarction patients, the Polyscore-based mortality prediction contained information additive to the conventional risk factors such as left ventricular systolic function and the Grace score. Intentionally, the Polyscore combined factors solely or largely related to the assessment of autonomic responsiveness.^17^^,^^18^ This makes the Polyscore-based risk prediction applicable to a broad spectrum of pathologies that affect the general homeostasis of the organism. This universality of the Polyscore contrasts with the concept of many other classification schemes that predominantly focus on a specific risk, such as the risk of cardiovascular complications.^3^

Hypothesizing that the Polyscore might contribute to general risk prediction in the elderly, we prospectively designed a sub-study of the INVADE population-based survey^6^ to validate the Polyscore in an elderly general population. Surprisingly, we found that even in such a cohort, where only 14% of patients had a history of coronary heart disease, the Polyscore is a powerful predictor of mortality. It is independent of other presently available risk characteristics: Two multivariable models, one including age, diabetes, chronic kidney disease, history of major stroke, and history of myocardial infarction, the other including the modified Framingham risk score, confirmed the independence of the Polyscore and clearly demonstrated its additive value. According to our results, the Polyscore effectively predicts all-cause mortality but not malignancy-related mortality, which might be less preventable than other forms of death.

In the population aged 60–74 years, the Polyscore-based risk prediction was particularly effective. This is consistent with the previously proposed hypothesis of autonomic reserve, which suggests that under normal physiologic conditions of younger subjects, autonomic maintenance of the organism can withstand moderate impacts and only becomes incapable of preserving the organism homeostasis when multiple negative impacts combine.^15^ It has repeatedly been reported that with advancing age, autonomic responsiveness/function declines.^16^ This is bound to decrease the predictive power of autonomic tests. In addition, different pathologies and comorbidities are more frequent at advanced age which leads to a higher (and expected) risk of death compared to the younger strata. The fact that the Polyscore does not stratify the mortality risk meaningfully in the population aged ≥75 years is thus not surprising. The reason why predictivity of the Polyscore is completely abolished in participants aged 75 years or older remains subject of speculation, but could be, in part, also a consequence of a smaller sample size.

Our practical experience suggests that screening of a general population of moderately advanced age, i.e. 60–75 years, would be fully feasible, even if the testing is performed repeatedly within this life span. The conduct of the necessary 30-min recordings requires only initiation and general supervision by trained support personnel. With reasonable quality of the recordings (as found largely in our data), we consider our algorithm suitable for a completely automated approach without any signal revisions. Shorter recording times have already been successfully tested for some individual parameters such as deceleration capacity and respiration rate^10^ but not for the complete Polyscore. This needs to be investigated in further follow-up studies.

Implementation of a comprehensive Polyscore screening program might advance preventive medical care substantially. Our results suggest that in such a scenario, further detailed clinical assessment could safely be delayed in apparently healthy subjects with low Polyscore values (i.e. in the low-risk category) who constitute the overwhelming majority of the population (almost 80% in our sample). In the remaining 20%, the intensity of immediate clinical evaluation may also be guided by the Polyscore values. Those with particularly high values (i.e. in the high-risk category) would require most detailed and urgent further evaluation, but this would still be realistic since such patients are infrequent (<5% in our sample).

For all these reasons, we strongly believe that a further prospective study is needed comparing the efficacy between the standard of care and a Polyscore-based approach in subjects aged 60–75 years. We trust that a new model of preventive healthcare tailored to the ageing population would emerge from such a prospective comparison.

Prospective nature of the present study needs to be stressed and highlighted. In the past, different retrospective risk prediction studies were published that used high-risk group definitions that were subsequently rejected in prospective validation. Although it would surely be possible to manipulate our data retrospectively to increase the predictive power of the individual Polyscore components and of their combination, such an approach would entirely invalidate the prospective proof of the previously established Polyscore definition^5^ that we present in this text.

Some limitations of the here presented study also need to be considered. Since concentrating on autonomic-based risk assessment and since using the predefined Polyscore, we have not considered other risk factors that could also be obtained from the available recordings (e.g. the QT interval duration and variability,^19^ QT/RR hysteresis, or the spatial QRS-T angle). The applicability to subjects in sinus rhythm excludes those in atrial fibrillation. Nevertheless, as atrial fibrillation patients receive increased medical attention, this is not a true limitation of the screening method. The source recordings of the Polyscore also identify patients with so far undiagnosed atrial fibrillation. We have not separated the population by sex although sex differences are known in autonomic indices of healthy subjects.^18^^,^^20^ The whole battery of tests included in the Polyscore assessment is based on the unprovoked recordings used in the study. There are other autonomic tests based on different provocations (e.g. Valsalva or head-up tilt). These were intentionally omitted when designing the Polyscore testing^5^ as they would require a more complex investigation setup. Since the Polyscore was designed to reflect a global autonomic evaluation, we considered only all-cause mortality in this investigation. The dichotomies of the Polyscore tests that were predefined and prospectively applied in this study were originally derived from studies of mortality risk prediction in cardiac patients.^11–14^ Possibly, different dichotomies might lead to more efficient risk stratification in the general population. Nevertheless, we have not performed any such investigation since it would have contradicted the prospective nature of the present study.

## Conclusion

Despite these limitations, the study not only shows that the Polyscore is a powerful predictor of mortality in the general population of moderately advanced age but also that risk prediction by the Polyscore is independent of other presently available risk characteristics. Using the Polyscore in a widespread regular screening programme of the population strata between 60 and 75 years of age would likely lead to a substantial preventive medicine improvement. Because of the simplicity of the unprovoked 30-min resting recordings, such a screening programme would be easy to implement.

## Supplementary material

Supplementary material is available at *Europace* online.

## Funding

This work was supported in part by the Allgemeine Ortskrankenkasse (AOK) Bayern and by the British Heart Foundation [New Horizons Grant NH/16/2/32499]. The sponsors had no input to the design or conduct of the study, to the evaluation of the results, to the writing of the report, and to the decision to submit the paper for publication.

**Conflict of interest:** G.S. holds patents covering the technology of some of the Polyscore tests. No other author of the manuscript has any personal, professional or financial relationships that could potentially be construed as a conflict of interest.

## Data availability

Source data of the study are available on request. Please contact alexander.steger@tum.de or gschmidt@tum.de.1

## Supplementary Material

euaa359_Supplementary_Data

## References

[1] GBD 2017 Mortality Collaborators. Global, regional, and national age-sex-specific mortality and life expectancy, 1950–2017: a systematic analysis for the Global Burden of Disease Study 2017. Lancet 2018;392:1684–735. 30496102 10.1016/S0140-6736(18)31891-9 PMC6227504

[2] Dieleman JL, Squires E, Bui AL, Campbell M, Chapin A, Hamavid H et al Factors associated with increases in US health care spending, 1996-2013. JAMA 2017;318:1668–78. 29114831 10.1001/jama.2017.15927 PMC5818797

[3] Collins GS, Altman DG. Predicting the 10 year risk of cardiovascular disease in the United Kingdom: independent and external validation of an updated version of QRISK2. BMJ 2012;344:e4181. 22723603 10.1136/bmj.e4181 PMC3380799

[4] Lind L, Sundstrom J, Arnlov J, Lampa E. Impact of aging on the strength of cardiovascular risk factors: a longitudinal study over 40 years. J Am Heart Assoc 2018;7:e007061. 29306895 10.1161/JAHA.117.007061 PMC5778963

[5] Steger A, Müller A, Barthel P, Dommasch M, Huster KM, Hnatkova K et al Polyscore of non-invasive cardiac risk factors. Front Physiol 2019;10:49. 30778303 10.3389/fphys.2019.00049 PMC6369149

[6] Scherpinski U, Bickel H, Gnahn H, Förstl H, Conrad B, Sander D. Interventionsprojekt zu zerebro- vaskulären Erkrankungen und Demenz im Landkreis Ebersberg (INVADE): rationale und Projektdesign. Nervenarzt 2002;73:1199–204. 12486573 10.1007/s00115-002-1443-8

[7] Schmidt G, Malik M, Barthel P, Schneider R, Ulm K, Rolnitzky L et al Heart-rate turbulence after ventricular premature beats as a predictor of mortality after acute myocardial infarction. Lancet 1999;353:1390–6. 10227219 10.1016/S0140-6736(98)08428-1

[8] Bauer A, Kantelhardt JW, Barthel P, Schneider R, Mäkikallio T, Ulm K et al Deceleration capacity of heart rate as a predictor of mortality after myocardial infarction: cohort study. Lancet 2006;367:1674–81. 16714188 10.1016/S0140-6736(06)68735-7

[9] Barthel P, Bauer A, Müller A, Huster KM, Kanters JK, Paruchuri V et al Spontaneous baroreflex sensitivity: prospective validation trial of a novel technique in survivors of acute myocardial infarction. Heart Rhythm 2012;9:1288–94. 22516186 10.1016/j.hrthm.2012.04.017

[10] Barthel P, Wensel R, Bauer A, Müller A, Wolf P, Ulm K et al Respiratory rate predicts outcome after acute myocardial infarction: a prospective cohort study. Eur Heart J 2013;34:1644–50. 23242188 10.1093/eurheartj/ehs420

[11] Sinnecker D, Dommasch M, Steger A, Berkefeld A, Hoppmann P, Müller A et al Expiration-triggered sinus arrhythmia predicts outcome in survivors of acute myocardial infarction. J Am Coll Cardiol 2016;67:2213–20. 27173032 10.1016/j.jacc.2016.03.484

[12] Sinnecker D, Dirschinger RJ, Barthel P, Müller A, Morley-Davies A, Hapfelmeier A et al Postextrasystolic blood pressure potentiation predicts poor outcome of cardiac patients. J Am Heart Assoc 2014;3:e000857. 24895163 10.1161/JAHA.114.000857 PMC4309081

[13] Anderson KM, Wilson PWF, Odell PM, Kannel WB. An updated coronary risk profile a statement for health professionals. Circulation 1991;83:356–62. 1984895 10.1161/01.cir.83.1.356

[14] Diener A, Celemín-Heinrich S, Wegscheider K, Kolpatzik K, Tomaschko K, Altiner A et al In-vivo-validation of a cardiovascular risk prediction tool: the arriba-pro study. BMC Fam Pract 2013;14:13. 23339773 10.1186/1471-2296-14-13 PMC3583804

[15] Barthel P, Bauer A, Müller A, Junk N, Huster KM, Ulm K et al Reflex and tonic autonomic markers for risk stratification in patients with type 2 diabetes surviving acute myocardial infarction. Diabetes Care 2011;34:1833–7. 21680727 10.2337/dc11-0330 PMC3142055

[16] Reardon M, Malik M. Changes in heart rate variability with age. Pacing Clin Electrophysiol 1996;19:1863–6. 8945057 10.1111/j.1540-8159.1996.tb03241.x

[17] Wellens HJ, Schwartz PJ, Lindemans FW, Buxton AE, Goldberger JJ, Hohnloser SH et al Risk stratification for sudden cardiac death: current status and challenges for the future. Eur Heart J 2014;35:1642–51. 24801071 10.1093/eurheartj/ehu176 PMC4076664

[18] Malik M, Hnatkova K, Huikuri H, Lombardi F, Schmidt G, Zabel M. Heart rate variability is a valid measure of cardiac autonomic responsiveness. J Physiol 2019;597:2595–8. 31006862 10.1113/JP277500 PMC6826215

[19] Baumert M, Porta A, Vos MA, Malik M, Couderc JP, Laguna P et al QT interval variability in body surface ECG: measurement, physiological basis, and clinical value. Europace 2016;18:925–44. 26823389 10.1093/europace/euv405 PMC4905605

[20] Ubrich R, Barthel P, Berkefeld A, Hnatkova K, Huster KM, Dommasch M et al Electrocardiographic and cardiac autonomic indices—implications of sex-specific risk stratification in women after acute myocardial infarction. Curr Pharm Des 2016;22:3817–28. 26965489 10.2174/1381612822666160311115605

