# Supplementary figures and images for: Polyscore of autonomic parameters for risk stratification of the elderly general population: the Polyscore study
Source: Europace. 2020 Dec 4;23(5):789–96. doi: 10.1093/europace/euaa359 (PMC8139819; doi:10.1093/europace/euaa359)

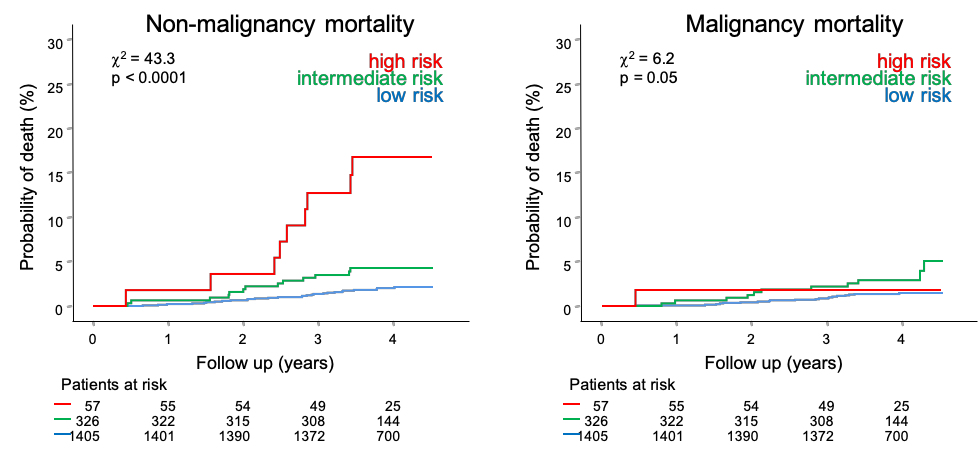

Supplement: euaa359_Supplementary_Data [file euaa359_supplementary_data.zip › Suppl Fig 1.jpg]

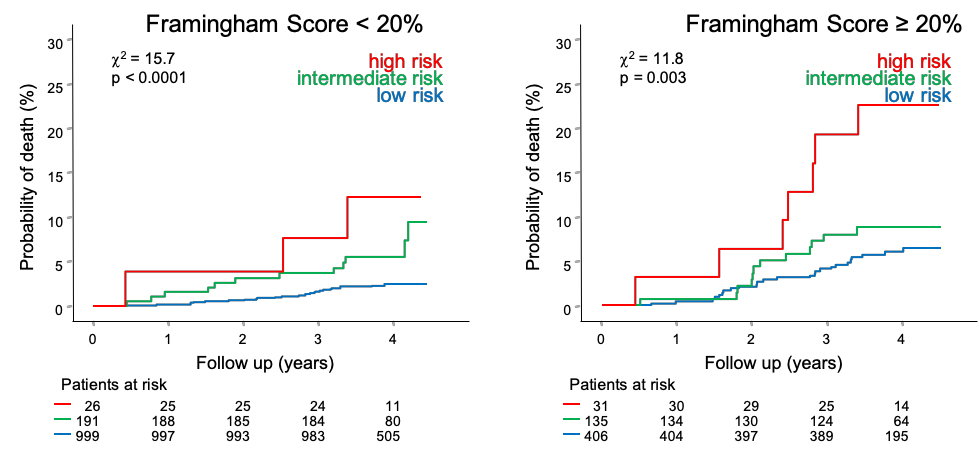

Supplement: euaa359_Supplementary_Data [file euaa359_supplementary_data.zip › Suppl Fig 2.jpg]
